# Effects of HPV16 E6 protein on Daxx-induced apoptosis in C33A cells

**DOI:** 10.1186/s11658-020-00230-z

**Published:** 2020-08-08

**Authors:** Shuangyang Tang, Shuang Ding, Lan Yu, Haiyan Shen, Yanping Wan, Yimou Wu

**Affiliations:** Pathogenic Biology Institute, University of South China, Hengyang, 421001 P. R. China

**Keywords:** Human papillomavirus type16, E6 protein, Death domain associated protein, Proliferation, Apoptosis, Caspase-8

## Abstract

**Aims:**

Daxx is a highly conserved nuclear protein with an important role in transcription, apoptosis and other cell processes. We investigated the role of HPV16 E6 in Daxx-induced apoptosis through their interactions in C33A cells.

**Methods:**

The binding of HPV16 E6 and Daxx was confirmed in C33A cells using co-immunoprecipitation and indirect immunofluorescence assays. Quantitative PCR and western blotting were performed to determine the RNA and protein expressions of Daxx, respectively. Automatic cell count and MTT assays were performed to investigate the proliferation of C33A cells. The apoptosis rate of C33A cells was determined via flow cytometry using Annexin V-FITC/PI staining. The relative activity of caspase-8 was tested using ELISA.

**Results:**

HPV16 E6 can bind with Daxx and cause its translocation in C33A cells. The transfected HPV16 E6 can cause a decrease in relative quantification for Daxx in Daxx-overexpressing cells. After Daxx transfection, cell proliferation was found to decrease sharply and cell apoptosis to increase sharply. However, when HPV16 E6 was co-transfected with Daxx, this decrease and increase both became gentle. Similarly, HPV16 E6 made the Daxx-induced increase in caspase-8 activity milder.

**Conclusions:**

HPV16 E6 is involved in inhibiting apoptosis through deregulation of Daxx-induced caspase-8 activities.

## Introduction

Death domain-associated protein (Daxx) is a highly conservative multi-function nucleoprotein that was first found in association with Fas-associated protein with death domain (FADD) as an activator of the cascade enzyme-related apoptosis [1]. Daxx can also participate in other cellular processes, including the mediation of protein interactions [2], promotion of cell apoptosis [3], restriction of virus replication [4] and induction of some viral genome chromatinization [5]. Daxx can reportedly also initiate an alternate apoptosis-mediating pathway involving the activation of apoptosis signal-regulating kinase 1 (ASK1) and c-Jun N-terminal kinase (JNK) [1, 6].

Human papilloma virus (HPV) infection causes a variety of epithelial lesions, including malignant cancers, such as cervical [7], vulvar [8] and anal [9] cancer. In fact, 50% of cervical cancer cases are closely associated with HPV16 [10]. HPV16 E6 protein is one of the oncoproteins coded by the HPV 16 early genome. It could play an important role in the development and progression of cervical cancer [11].

Our preliminary studies found that HPV16 E6 can interact with Daxx, which can lead to the translocation of Daxx in HeLa cells (an HPV 18-transformed cervical cancer cell line that contains disrupted copies of the E2 gene) [12]. Since Daxx can be involved in cell apoptosis, it is possible that the interaction of these two proteins might interrupt Daxx-mediated apoptosis.

This study confirms the effects of HPV16 E6 and Daxx on apoptosis. We also show that caspase-8 activity relies on the Daxx–JNK signal pathway, which will help elucidate the role of Daxx in the occurrence and development of cervical cancer with associated HPV16 infection. It could also offer a basis for research into the treatment of HPV16-positive cervical cancers.

## Material and methods

### Plasmids and reagents

The *pcDNA3.1(+)/HPV16 E6* and *pcDNA3.1(+)/Daxx* plasmids were provided by the Institute of Pathogenic Biology of the University of South China. The AxyPrep Maxi Plasmid Kit was purchased from Axygen Biosciences (USA). Rabbit anti-human Daxx antibody and mouse anti-human HPV16 E6 antibody were purchased from Santa Cruz (USA). HRP-Goat Anti-Rabbit IgG and HRP-goat anti-mouse IgG, anti-GAPDH mouse monoclonal antibody, FITC-goat anti-rabbit IgG and TRITC-goat anti-mouse IgG were all obtained from Sigma (USA). Lipofectamine 2000 and thiazolyl blue tetrazolium bromide (MTT) were purchased from Invitrogen (USA). C33A cells, representing an HPV-negative squamous cervical cancer cell line, were purchased from the ATCC (USA). Caspase 8 Activity Colorimetric Assay Kit and Annexin V-FITC Apoptosis Detection Kit were purchased from MultiSciences (Lianke) Biotech (China).

### Cell transfection

The C33A cells were cultured in Dulbecco’s modified Eagle medium (DMEM) containing 10% fetal bovine serum (FBS) (40 g/l) at 37 °C with 5% CO_2_. When the cell confluence reached 50%, the growth solution was discarded and the cells were washed twice with basic DMEM. Prior to this, Lipofectamine 2000 had been mixed with *pcDNA3.1*(+)*/HPV16 E6* and/or *pcDNA3.1*(+)*/Daxx* plasmid in DMEM for 15 min. This mixture was added to DMEM with the washed cells. After 6 h of culture at 37 °C with 5% CO_2,_ the basic DMEM was replaced with DMEM supplemented with 10% FCS for further culture.

### Co-immunoprecipitation test

C33A cells (1 × 10^5^ cells/ml) were added to 24-well plates (1 ml/well). The *pcDNA3.1(+)/HPV16 E6* transfection was done after 18 h. Culture ran for a further 48 h, then the cells were washed twice with cool phosphate-buffered saline (PBS) and dissolved under slow rotation at 4 °C for 30 min. After centrifugation, the lytic supernatant of the cell lysate was mixed with anti-Daxx or anti-E6 antibodies. The mixture was incubated at 4 °C overnight. Protein A/G agarose was added and the mixture was rotated at 4 °C for 3 h, then centrifuged. The precipitate was washed 4 times with 1 ml of lysate buffer, then mixed with a sodium dodecyl sulfate (SDS) sample buffer. This mixture was heated to boiling and the supernatant was obtained by centrifugation for SDS polyacrylamide gel electrophoresis (SDS-PAGE).

For the western blot assay (WB), each sample was divided into two: one with anti-Daxx as the primary antibody, the other with anti-E6. The respective positive controls for Daxx and HPV16 E6 were the lytic supernatants of C33A cells with anti-Daxx or anti-E6 antibody. IgG antibody was used as the negative control.

### Indirect immunofluorescence assay

C33A cells (1 × 10^5^ cells/ml) were added to 24-well plates (1 ml/well) with round glass sheets in each well. Transfection proceeded for 48 h as described above with five groups: blank (C33A cells without transfection), negative control (transfected with *pCDNA3.1(+)-C1* empty plasmid), Daxx (transfected with *pCDNA3.1(+)/Daxx*), Daxx+E6 (co-transfected with *pCDNA3.1(+)/Daxx* and *pCDNA3.1(+)/HPV16 E6*), and E6 (transfected with *pCDNA3.1(+)/E6*). After transfection, the cell growth solution was discarded and the cells were washed twice with PBS. After fixation with 4% triformol, perforation with 0.2% Triton X-10 and blocking with 2% bovine serum albumin (BSA), overnight incubation with anti-Daxx and anti-E6 antibodies was performed. A mixture of FITC-GAR, TRITC-GAM and DAPI was added at 37 °C for 1 h, and after the last wash, the supernatant was discarded. Finally, the round glass sheets were taken out and sealed on slides with anti-fluorescence quencher. The cells were observed under a fluorescence microscope.

### Real-time quantitative PCR

A culture of 1 × 10^5^ C33A cells/ml in 6-well plates (2 ml/well) was allowed to reach the 50–70% confluence. Then, transfection proceeded for 48 h. The culture supernatants were discarded and RNA samples were prepared from the collected cells with the addition of TRIZOL. RNA samples from the various transfected cells were used independently for the validation experiments. The quantitative PCR assay was performed using SYBER Select Master Mix (Applied Biosystems, USA). The housekeeping gene GAPDH was expressed and used as a normalizing control. Relative quantification of Daxx was evaluated using the double delta Ct (ΔΔCt) method.

### Western blot assay

The transfected C33A cells were cultured in 6-well plates for 48 h, then the culture supernatants were discarded and the cells were washed twice with PBS. The lytic supernatant of the cell lysate was dissolved with the addition of cell lysis solution under slow rotation at 4 °C for 30 min and then collected via centrifugation. After the protein concentration of the prepared lysate was tested according to the method described above, the protein lysate was mixed with SDS sample buffer and prepared for SDS-PAGE. GAPDH was used to as the internal reference protein, and its corresponding antibody was used to test its expression. Similarly, the anti-Daxx antibody was used to test the expression of Daxx protein.

### Automatic cell count assay

We added 1 × 10^5^ C33A cells/ml to 6-well plates (2 ml/well). Transfection proceeded as described above. After 48 h, the cells were suspended in 1 ml DMEM and 1 μl cell suspension was removed for cell counting in BodBoge Automatic Cell counter. Viable cells numbers were compared for each group.

### MTT detection for cell proliferation inhibition

We added 1 × 10^5^ C33A cells/ml to 96-well plates (200 μl/well). After 44 h culture, 20 μl MTT was added to each well. Culture continued for 4 h. Then, the culture supernatant was discarded vua centrifugation, and 150 μl DMSO was added. The plates were shocked for 15 min and the OD_450_ values were measured. The effects of HPV16 E6 on C33A cells were analyzed using the proliferation inhibition (PI) ratio:

$$ 1-\left({\mathrm{OD}}_{450}\mathrm{of}\ \mathrm{tested}\ \mathrm{group}/{\mathrm{OD}}_{450}\mathrm{of}\ \mathrm{control}\ \mathrm{group}\right)\times 100\% $$

### Fluorescence detection for apoptosis morphology

We added 1 × 10^5^ C33A cells/ml to 24-well plates with round glass sheets in them (1 ml/well) and to 6-well plates (2 ml/well). Transfection proceeded as described above. After 48 h, the round glass sheets were fixed with fixative for 10 min, washed twice with cooling PBS, stained with Hoechst 33258, then washed twice with cooling PBS again. Finally, they were taken out to be sealed on slides with anti-fluorescence quencher and observed under Nikon fluorescence microscope.

### Flow cytometry for apoptosis detection

Transfection proceeded in 6-well plates as described above for 48 h. Then, the transfected C33A cells were collected, washed twice with cooling PBS and re-suspended in 50 μl sample buffer. Then 5 μl Annexin V-FITC and 2.5 μl propidium iodide were added to the re-suspended cells for staining. After 10 min of incubation without light, the apoptotic cells were detected using flow cytometry (FCM).

### Spectrophotometry for determining the relative activity of caspase-8

After 48 h transfection in 6-well plates, the cells were dissolved via shaking with Solarbio cell lysis solution on ice for 10 min. As per the kit instructions, over 50 μl cell lysis solution and 200 μg protein were added to 2 x reaction buffer. The mixture was mixed with 5 μl caspase-8 substrate and incubated without light at 37 °C for 4 h. Finally, the absorbance value under 405 nm wavelength (A_405_) was read using an Amersham spectrophotometer. The A_405_ of the blank control (50 μl cell lysis solution without any cells) was taken as zero. The A_405_ of the other wells was taken as the relative activity of caspase-8 in those wells.

### Statistical analysis

The data are shown as means ± standard deviation ($\overline{x}$ ±s), which were compared using the one-way ANOVA method and analyzed using statistics software SPSS18.0. A value of *p* < 0.05 was taken as statistically significant.

## Results

### Interaction of HPV16 E6 and Daxx in C33A cells

**Fig. 1:**
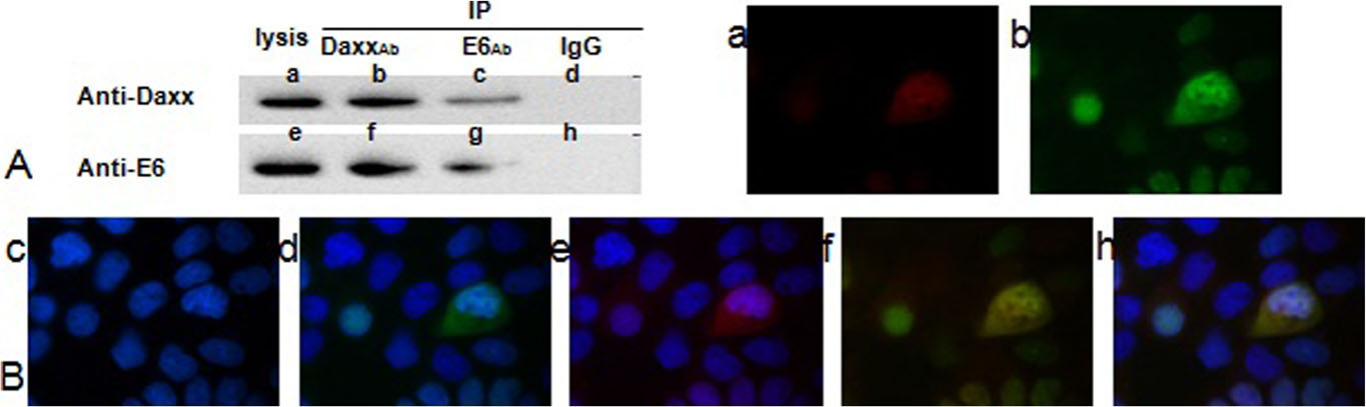
Interaction of HPV16 E6 and Daxx in C33A cells. **A** Binding of HPV16 E6 and Daxx assessed using western blotting. Photo shows representative blots for: (a) the cell lytic supernatant with anti-Daxx antibody (positive control for Daxx); (b) the complex of Daxx pulled down by the anti-Daxx antibody with anti-Daxx antibody; (c) the complex of Daxx pulled down by the anti-E6 antibody with anti-Daxx antibody; (d) the complex pulled down by the IgG antibody with anti-Daxx antibody (negative control of Daxx); (e) the cell lytic supernatant with anti-E6 antibody (positive control of HPV16 E6); (f) the complex pulled down by the anti-Daxx antibody with anti-E6 antibody; (g) the complex pulled down by the anti-E6 antibody with anti-E6 antibody; (h) the complex pulled down by the IgG antibody with anti-E6 antibody (negative control of HPV16 E6). **B** Localization of HPV16 E6 and Daxx via indirect immunofluorescence. (a) C33A cells with mouse anti-E6 antibodies visualized with TRITC-conjugated goat anti-mouse IgG (red); (b) C33A cells with rabbit anti-Daxx antibodies visualized with FITC -conjugated goat anti-rabbit IgG (green); (c) C33A cells with DNA dye (blue); (d) a and c overlapped; (e) b and c overlapped; (f) a and b overlapped, showing yellow fluorescence; (h) a, b and c overlapped

The positive controls (complexes of E6 with anti-E6 antibody or Daxx with anti-Daxx antibody) could be detected from the lytic supernatant of C33A cells transfected with *pcDNA3.1(+)/HPV16 E6* or *pcDNA3.1*(+)*/Daxx*. Anti-E6 antibody could detect a complex precipitated out by anti-Daxx antibody or anti-E6 antibody. Similarly, anti-Daxx antibody could detect a complex precipitated out through anti-Daxx antibody or anti-E6 antibody (Fig. 1A).

Blue fluorescence was assigned to the nucleus. The red fluorescence of HPV16 E6 was distributed in the nucleus and cytoplasm. The green fluorescence of Daxx was mainly in the nucleus. However, in the cells expressing HPV16 E6, the green fluorescence was distributed in the nucleus and cytoplasm too. Superimposition of the images revealed the yellow fluorescence (Fig. 1B).

### Effects of HPV16 E6 on Daxx expression

**Fig. 2:**
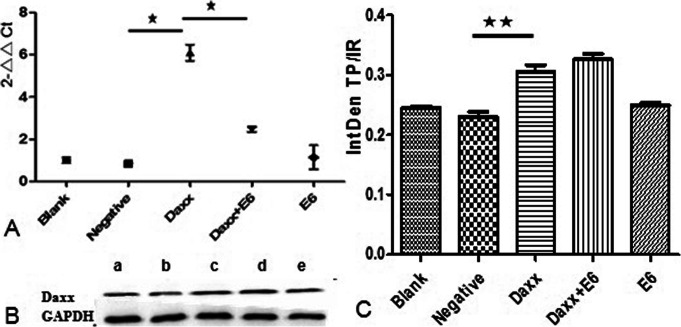
Effects of HPV16 E6 on Daxx expression. **A** Relative quantification of Daxx RNA via quantitative PCR. Relative quantification of Daxx was evaluated using the double delta Ct (ΔΔCt) method. ★, *p* < 0.05. **B** Daxx protein expression evaluated via western blotting. (a) C33A cells without transfection; (b) C33A cells with empty vector transfection; (c) C33A cells with Daxx transfection; (d) C33A cells with E6 and Daxx co-transfection; (e) C33A cells with E6 transfection. **C** Integrated density values based on western blotting results. IntDen TP/IR describes the ratio of the integrated density of the internal reference to that of the target protein (IntDen IR/TP). ★★, *p* < 0.01

As shown in Fig. 2A, there was not much statistical difference in the relative quantification for Daxx RNA between the Negative and Blank groups (*p* > 0.05), but there was a significant difference between the Daxx and Negative groups, as expected (*p* < 0.01). There was not much statistical difference between the E6 and Negative groups (*p* > 0.05). However, there was a clear decrease from the single Daxx transfection to the co-transfection group, and the differences between the Daxx+E6 and Daxx groups was statistically significant (*p* < 0.05). This suggests that HPV16 E6 may have some influence on the function of Daxx.

As shown in Fig. 2B and C, the differences in protein expression of Daxx between the Negative and Blank groups (*p* > 0.05) and the Daxx and Negative groups (*p* < 0.01) were similar to those for the relative quantification of Daxx RNA, showing that *pCDNA3.1(+)/Daxx* was successfully transfected into C33A cells.

### Effects of HPV16 E6 on the proliferation of C33A cells

**Fig. 3:**
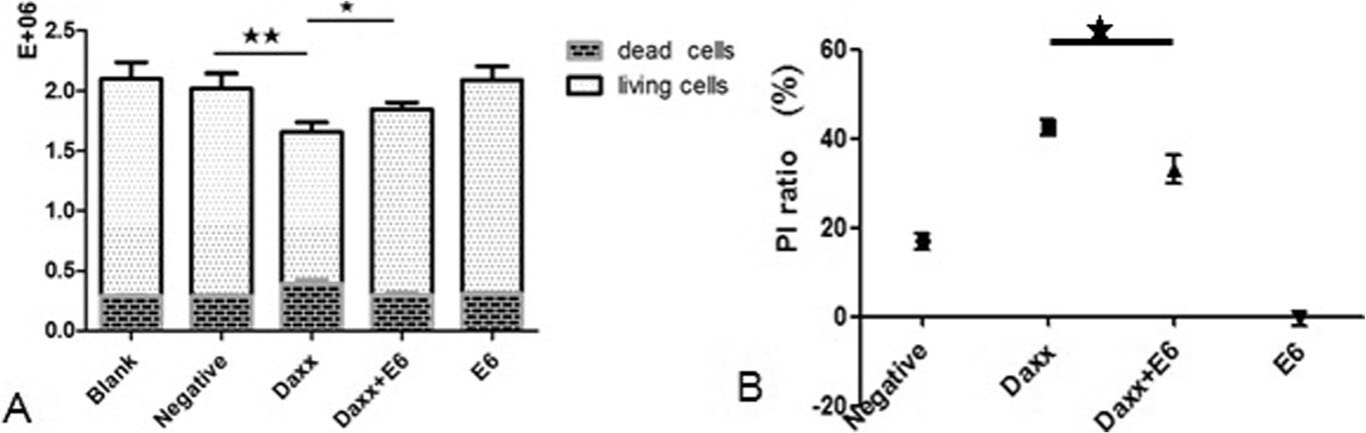
Effects of HPV16 E6 on cell proliferation. **a** Cell count results for all groups. The cell counting unit was 10^6^, i.e., E+ 06. **b** Proliferation inhibition ratios for all groups. The proliferation inhibition (PI) ratio represents the inhibition of cell proliferation. ★, *p* < 0.05; ★★, *p* < 0.01

As shown from the cell count results (Fig. 3a), the differences in total cell number, dead cell number or viable cell number between the Daxx-transfected group and the negative control were statistically significant (*p* < 0.05). However, the differences between the HPV16 E6-transfected group and negative group were not statistically significant (*p* > 0.05). Moreover, the difference in viable cell number for the Daxx-transfected group was clearly lower than that for the HPV16 E6 and Daxx co-transfected group (*p* < 0.05).

The MTT tests (Fig. 3b) showed that the cell proliferation in the Daxx-transfected group was clearly lower than that in the negative control group (*p* < 0.01). The PI ratio for the Daxx-transfected group was higher than that for the other groups and these differences were also statistically significant (*p* < 0.01). The difference between the HPV16 E6-transfected and negative groups was not statistically significant (*p* > 0.05). Importantly, the difference between the Daxx-transfected group and HPV16 E6 and Daxx co-transfected group was also not statistically significant (*p* > 0.05), indicating that HPV16 E6 may be not enough to inhibit the negative regulation of Daxx on cell proliferation.

### Effects of HPV16 E6 on the apoptosis of C33A cells

**Fig. 4:**
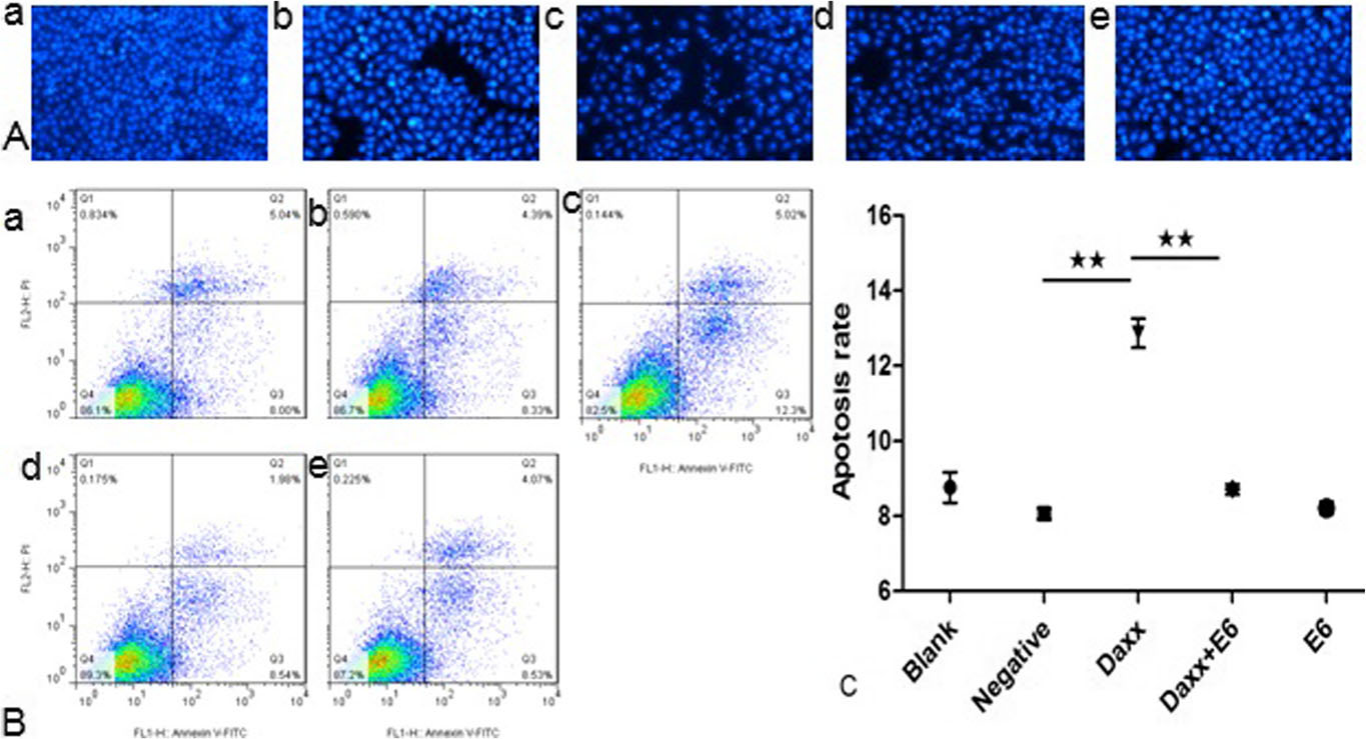
Effect of HPV16 E6 on apoptosis of C33A cells. **A** Observation of representative apoptotic cells. The groups with Daxx transfection had more apoptotic cells than the other groups. (a) C33A cells without transfection; (b) C33A cells with empty vector transfection; (c) C33A cells with Daxx transfection; (d) C33A cells with E6 and Daxx co-transfection; (e) C33A cells with E6 transfection. **B** Representative flow cytometry data. (a) C33A cells without transfection; (b) C33A cells with empty vector transfection; (c) C33A cells with Daxx transfection; (d) C33A cells with E6 and Daxx co-transfection; (e) C33A cells with E6 transfection. **C** Apoptosis rate. The mean apoptosis rate of each group came from the flow cytometry data. ★★, *p* < 0.01

The apoptotic cells were observed intuitively under a fluorescence microscope (Fig. 4A). It was clear that the groups with Daxx transfection had more apoptotic cells than the other groups. The characteristic morphological changes of apoptosis were observed in cells that had undergone Daxx treatment.

As expected, the results of the FCM tests (Fig. 4B and C) showed that there was not much difference between the groups with empty plasmid transfection and non-transfection statistically (*p* > 0.05). Similarly, the difference between the HPV16 E6-transfected group and the empty plasmid-transfected group was not statistically significant (*p* > 0.05), indicating that HPV16 E6 had little effect on the apoptosis of C33A cells. There was considerable difference between the groups with Daxx transfection and with empty plasmid transfection (*p* < 0.01), showing that Daxx transfection caused more apoptosis. However, it was found that there was a significant difference between the Daxx and HPV16 E6 co-transfected group and the Daxx-transfected group (*p* < 0.01).

Summing up, there was no obvious decrease or increase in apoptosis of C33A cells after HPV16 E6 transfection (*p* > 0.05). It was also evident that the apoptosis of C33A cells with Daxx and E6 co-transfection was low compared with that of cells with Daxx transfection alone. It is thus unknown whether HPV16 E6 can inhibit or promote cell apoptosis, but it can clearly affect the apoptosis caused by Daxx. This may be related to the interaction of HPV16 E6 and Daxx.

### Impact of HPV16 E6 on caspase-8 activity in C33A cells

**Fig. 5:**
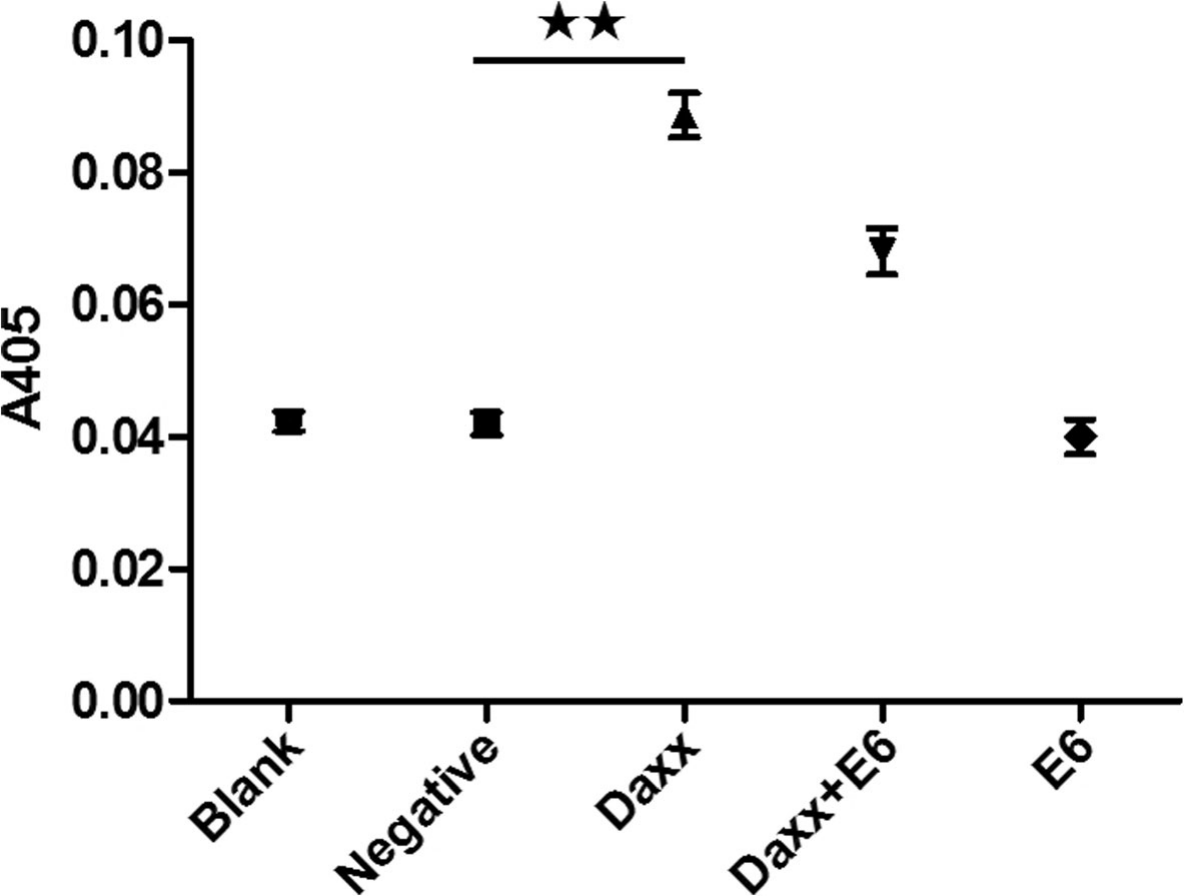
Effects of HPV16 E6 on caspase-8 activity. The A405 value represents the relative activity of caspase-8. ★★, *p* < 0.01

As shown in Fig. 5, the caspase-8 activity of the Daxx-transfected group was higher than that of the empty plasmid-transfected group, and this difference had statistical significance (*p* < 0.01). However, there was no difference between the HPV16 E6-transfected group and the negative control group (*p* > 0.05). Moreover, the caspase-8 activity of the co-transfected group was statistically significantly higher than that of the HPV16 E6*-*transfected group (*p* < 0.01). However, the differences between the Daxx-transfected group and co-transfected group were not statistically significant (*p* > 0.05).

## Discussion

HPV16 E6 is a major protein involved in the transformation of malignant cells. It can interact with various signaling molecules, including p53, P300, E6AP, hADA3, Gps2, Bak, TNFR, FADD, caspase-8 and hMCM7 [13]. This affects signaling pathways, cell microenvironments, virus life cycle and host cell biological functions. It can promote the immortalization of host cells.

In cervical cancer cells, HPV16 E6 oncoprotein can suppress p53 protein [14]. In human cervical keratinocyte line (HCK1T) cells, the expression of HPV16 E6 can result in reduced p53 production [15]. This facilitates HPV16 infection and can even promote the development and progression of some cancers, including cervical cancer [11] and vulvar carcinoma [16].

PML-NBs are relevant for HPV E1, E2, E5, E6, E7 and L1 [17]. Daxx can interact with some viral oncoproteins with a change in cellular localization, resulting in a change in biological function. Preliminary results found that HPV16 E6 could interact with Daxx, leading to its translocation in HeLa cells and suggesting that this interaction may affect its normal function. To confirm the interaction of HPV16 E6 and Daxx in C33A cells, we investigated the effects of Daxx on cell apoptosis in the presence of HPV16 E6.

The stability of PML-NBs plays a critical role in the immune response and tumor suppression [18]. When PML-NBs are absent or disrupted, their function can change and even become contradicted. It was reported that Daxx could induce ovarian cancer ascite formation by activating the ERK signal pathway and binding to CEBP-β [19], but this may be because Daxx is a mutant in most pancreatic neuroendocrine tumors [20]. The N-terminal death effector domain (DED) of FADD can bind with the precursor protein of caspase-8 (or − 10) and form the death-inducing signaling complex (DISC) to activate caspase-8 (or 10), further activating the caspase cascade and leading to cell apoptosis [21]. Daxx can associate with FADD and activate the cascade enzyme to induce apoptosis [5]. Although there was a report that Daxx cannot mediate Fas-induced apoptosis [22], other researchers considered that, owing to the increased signals in cleaved caspase-8, Fas–Daxx interaction can have a proapoptotic effect in mouse motor neuron-neuroblastoma hybrid cells [23].

However, HPV16 E6 protein can bind the site of the N-terminal in DED [24] to induce FADD degradation, which in turn may block the proenzyme of caspase-2, − 8, − 9 and − 10 gathering near FADD. Therefore, HPV16 E6 can compete with the precursor protein of caspase-8 to bind with the DED of FADD, thus inhibiting caspase activation. Similarly, the binding of HPV16 E6 with FADD may affect the association of Daxx with FADD, as may the interaction of HPV16 E6 with Daxx, which can decrease the activation of cascade enzyme, resulting in a decrease in Daxx-induced apoptosis.

It was reportedly found that the transfection of HPV16 E6 to HCT116 cells could suppress the apoptosis induced by Fas, and then promote procaspase-8 degradation, followed by inhibition of the activation of caspase-8, − 3 and − 2 [25]. Similarly, it was found that caspase-8 expression decreased in the skin of transgenic mice with HPV16 E6 [26]. However, Manzo-Merino reported that HPV16 E6 expression could increase caspase-8 activation, even with a slight reduction in the total levels of caspase-8 expression, but that it did not result in an increase in apoptosis induced by caspase-8 in HEK293 cells [27]. Blocking HPV16 E6 or caspase-8 binding reportedly increases the level of caspase-8 and p53 in cervical cancer SiHa cells, resulting in an increase in caspase-3 and -7 activity, but this was not effective in HPV-negative cervical or oral cancer cells [28].

Therefore, HPV16 E6 had no distinct effects on caspase-8 activity, indicating that the influence of HPV16 E6 on the binding of FADD with the precursor protein of caspase-8 was not obvious in this study. However, when compared with the results for the HPV16 E6-transfected group, the increase in caspase-8 activity in co-transfected group may be explained by the transfected Daxx potentially blocking the HPV16 E6 or caspase-8 binding. This could result in a release of caspase-8 activity, indicating that when there is sufficient caspase-8, the interaction of HPV16 E6 and caspase 8 may affect the cascade enzyme and affect cell apoptosis.

It was reported that when Daxx existed in the cytoplasm and nucleus in matrix protein mutant soluble tumor cells with vesicular stomatitis virus infection, Fas-mediated caspase activation relied on a Daxx–JNK signal rather than an FADD signal [29]. Likewise, when it was found that HPV16 E6 interacted with Daxx, leading to Daxx being translocated from the nucleus to cytoplasm, it was thought that the interaction of HPV16 E6 and Daxx might affect the activation of the Daxx–JNK signaling pathway and caspase-8 activation, eventually leading to an inhibition of cell apoptosis. In this study, we found that the transfected HPV16 E6 can downregulate the increasing activation of caspase-8 caused by Daxx transfection in cervical cancer C33A cells.

Thus, we have shown that Daxx can promote cell apoptosis through regulation of caspase-8 activation in C33A cells and that HPV16 E6 can deregulate Daxx-induced caspase-8 activation and pro-apoptosis. This means that HPV16 E6 is involved in inhibiting cell apoptosis through deregulating Daxx-induced caspase-8 activation and pro-apoptosis. The specific mechanism needs further study. Our results still offer experimental evidence for Daxx being a promising target for intervention therapy in HPV16-positive cervical cancer.

## Data Availability

Not applicable.

## Abbreviations

ASK1: Apoptosis signal-regulating kinase 1
Co-IP: Co-immunoprecipitation
Daxx: Death domain-associated protein
DED: N-terminal death effector domain
DISC: Death-inducing signaling complex
DMEM: Dulbecco’s modified Eagle medium
FBS: Fetal bovine serum
FCM: Flow cytometry
FADD: Fas-associated protein with death domain
HPV: Human papilloma virus
MTT: Thiazolyl blue tetrazolium bromide
PBS: Phosphate-buffered saline
SDS-PAGE: Sodium dodecyl sulfate polyacrylamide gel electrophoresis
WB: Western blot assay
